# Amebicidal and Antiadhesion Activities of *Knema retusa* Extract Against *Acanthamoeba triangularis* T4 Genotype on Contact Lenses and Modeling Simulation of Its Main Compound, E2N, Against *Acanthamoeba* Beta-Tubulin

**DOI:** 10.1155/sci5/4311313

**Published:** 2025-01-30

**Authors:** Watcharapong Mitsuwan, Imran Sama-ae, Suthinee Sangkanu, Dhrubo Ahmed Khan, Partha Biswas, Md. Nazmul Hasan, Julalak Chuprom, Tajudeen O. Jimoh, Christophe Wiart, Masyitah Binti Zulkipli, Nor Hayati Abdullah, Maria de Lourdes Pereira, Sonia M. Rodrigues Oliveira, Shanmuga Sundar Saravanabhavan, Polrat Wilairatana, Tooba Mahboob, Veeranoot Nissapatorn

**Affiliations:** ^1^Akkhraratchakumari Veterinary College, Walailak University, Nakhon Si Thammarat, Thailand; ^2^One Health Research Center, Walailak University, Nakhon Si Thammarat, Thailand; ^3^Center of Excellence in Innovation of Essential Oil, Walailak University, Nakhon Si Thammarat, Thailand; ^4^School of Allied Health Sciences, Southeast Asia Water Team (SEA Water Team), World Union for Herbal Drug Discovery (WUHeDD), Research Excellence Center for Innovation and Health Products (RECIHP), Walailak University, Nakhon Si Thammarat, Thailand; ^5^Department of Genetic Engineering and Biotechnology, Faculty of Biological Science and Technology, Jashore University of Science and Technology, Jashore, Bangladesh; ^6^School of Languages and General Education, Walailak University, Nakhon Si Thammarat, Thailand; ^7^Department of Microbiology, Icahn School of Medicine at Mount Sinai, New York, New York, USA; ^8^The Institute for Tropical Biology and Conservation, Universiti Malaysia Sabah, Jalan UMS, Kota Kinabalu, Sabah, Malaysia; ^9^School of Pharmacy, University of Nottingham Malaysia Campus, Semenyih, Malaysia; ^10^Natural Product Division, Forest Research Institute Malaysia (FRIM), Kepong, Malaysia; ^11^CICECO-Aveiro Institute of Materials, University of Aveiro 3810-193, Aveiro, Portugal; ^12^Department of Medical Sciences, University of Aveiro 3810-193, Aveiro, Portugal; ^13^Faculty of Dental Medicine, Center for Interdisciplinary Research in Health (CIIS), Universidade Católica Portuguesa 3504-505, Viseu, Portugal; ^14^Department of Biotechnology, Aarupadai Veedu Institute of Technology, Vinayaka Mission's Research Foundation (DU), Paiyanur, Chennai, India; ^15^Department of Clinical Tropical Medicine, Faculty of Tropical Medicine, Mahidol University, Bangkok, Thailand; ^16^Faculty of Pharmaceutical Sciences, UCSI University, Kuala Lumpur, Malaysia

**Keywords:** β-tubulin, *Acanthamoeba triangularis*, anti-*Acanthamoeba* and antiadhesion activities, contact lenses, *Knema retusa*, molecular dynamics simulation

## Abstract

Medicinal plants have been used as alternative agents for the treatment of infections. This study aimed to investigate bioactivities of medicinal plant extracts including *Knema retusa* extract (Kre) against *Acanthamoeba triangularis* T4 *in vitro* and *in silico*. Anti-*Acanthamoeba* activities of 44 extracts from 5 plant species were determined. From 44 tested extracts, a chloroform extract of Kre bark showed the strongest anti-*Acanthamoeba* activities against both trophozoites and cysts, with MIC values of 32.25 and 62.50 μg/mL, respectively. Then, amebicidal and antiadhesion activities of Kre against *A. triangularis* were investigated. Kre reduced the growth by 3 logs within 8 h at 4 × MIC. Disruption of the cells with abnormal shapes was observed when trophozoites were treated with Kre. Trophozoites had lost their robust acanthopodia and began to shrink after treatment with Kre. Treated cysts exhibited wall disruption and dramatically showed forms of marked retraction. Treatment of Kre at 1/2 × MIC showed about 87% reduction in the trophozoite adhesion, while treatment at 2 × MIC exhibited a 59% reduction in the trophozoite adhesion to the plastic surface, compared with the control. Furthermore, 1 log cells/mL (90%) of the contact lens adhesive trophozoites were reduced and removed after treatment with Kre. Molecular docking indicated that E2N, the main compound in Kre, exhibited strong binding to the ligand binding sites at β-tubulin, with a binding energy of −7.01 kcal/mol and an inhibitory constant of 2.43–7.32 μM. E2N generated multiple connections via hydrogen, hydrophobic, ionic, and water bridge bonding and maintained these connections until the simulation finished, facilitating the creation of stable bindings with the β-tubulin protein as measured by molecular dynamics simulation. These findings suggest that Kre exhibits amebicidal and antiadhesion activities which could be used for the prevention of *A. triangularis* adhesion to contact lenses.

## 1. Introduction

*Acanthamoeba triangularis* (*A. triangularis*) is a free-living protozoan that can be found in nature, including soil and water. The parasite belongs to the genus *Acanthamoeba* and is considered a human pathogen [1]. The parasite develops in two stages, including trophozoites and cysts. Trophozoites are a type of vegetative amoeba that move using amoeboid locomotion. The cyst form is a latent stage that can endure harsh environmental circumstances, such as lack of nutrients. However, *A. triangularis* causes granulomatous amoebic encephalitis and *Acanthamoeba* keratitis in healthy humans and immunocompromised hosts. Serious vision loss and complete blindness caused by the parasites are the most frequent keratitis instances among contact lens wearers [2]. Contact lenses are therefore thought to be the primary risk factor for the spread of *A. triangularis* to the eyes. A critical early step in the development of keratitis is displayed by *Acanthamoeba* attachment to the host cells [3]. In addition, contact lenses can be adhered to by *Acanthamoeba* trophozoites using acanthopodia, which are thorn-like projection pseudopodia [3]. Hence, removal of the parasite from the human body and the contact lenses is difficult due to the presence of the acanthopodia in the parasite. Moreover, treatment of *Acanthamoeba* infections is difficult due to its double-walled cyst layers, including the ectocyst and endocyst walls [4].

Novel metabolites from medicinal plants have been used as an alternative treatment for the management of protozoa associated infection such as *A. triangularis*. This could be correlated with previous studies from [1, 5] where an ethanolic extract from *Curcuma longa* rhizome exhibited anti-*Acanthamoeba* activity against *A. triangularis*. Also, a significant decrease in the adhesion of *A. triangularis* to contact lenses via acanthopodia disruption from *C. longa* extract and its potent compound curcumin was reported in [5]. The present study focused on medicinal plants including *Burkillanthus malaccensis*, *Cleistanthus bracteosus*, *Diospyros hasselti*, *Knema retusa*, and *Litsea spathacea*. Those plant species are located in Manong and Kuala Kangsar, State of Perak, Malaysia, and well known as the medicinal plants. Interestingly, a wood extract of *Knema retusa*, a plant belonging to the Myristicaceae family [6], showed antibacterial and antibiofilm activities against *Staphylococcus aureus* and *Staphylococcus haemolyticus* isolated from bovine mastitis [7]. For folkloric employment, some *Knema* species have been used the treatment of infectious diseases [6]. It has been reported that endo-2-hydroxy-9,9-(ethylenedioxy)-1-carbethoxy bicyclo [3.3.1] nonane (E2N) is the major ingredient of Kre [7]. According to molecular docking, E2N had a strong affinity for staphylococcal accessory regulator A (SarA), a key regulatory component that regulates the synthesis of staphylococcal virulence factors [7].

Therefore, this study sought to investigate the anti-*Acanthamoeba* activity of Kre against trophozoites and cysts of *A. triangularis*. The synergistic effects of the plant extract in combination with antibiotics were investigated. The effects of Kre on the adhesion of *A. triangularis* to the plastic surface and contact lenses were carried out. *In silico* approaches including molecular docking and molecular dynamics simulation (MDS) of E2N to *Acanthamoeba* tubulin were investigated. Tubulin containing alpha and beta tubulin is a monomer of microtubules that form a part of eukaryotic cells and are key components of the cytoskeleton. Tubulin plays a crucial role in chromosomal segregation, organelle movement, and cellular motility. Moreover, tubulin is involved in the preservation of cell shape in organisms, including *Acanthamoeba* spp. [8]. Interestingly, targeting the process of microtubule dynamics is an excellent strategy for chemotherapy, and inhibiting microtubule dynamics is recognized as one of the most effective strategies for treating cancer [9] as well as the infection caused by *Acanthamoeba* spp. [9]. Therefore, inhibition of tubulin by antimicrobial compounds may be an alternative strategy to inhibit the growth of *Acanthamoeba* spp. including *A. triangularis*.

## 2. Materials and Methods

### 2.1. Preparation of Medicinal Plant Extracts and Antimicrobial Agents

Each part of five Malaysian medicinal plants including *Burkillanthus malaccensis*, *Cleistanthus bracteosus*, *Diospyros hasselti*, *Knema retusa*, and *Litsea spathacea* (Table 1) was collected from Manong and Kuala Kangsar, State of Perak, Malaysia (4°7′ North, 100°8′East). The plant species was identified by botanists at the National Herbarium of the Forest Research Institute of Malaysia (FRIM). Plant parts used and solvents (chloroform, hexane, and methanol) are presented in Table 1. The plant materials were air-dried at room temperature for 2 weeks and then finely pulverized by grinding using an aluminum collection blender (Philips, Shanghai, China). Dried plant powders (200 g) were soaked at room temperature with different solvents (1:5 powder: solvent, w/v) for 3 days. The respective liquid extracts were subsequently filtered through Whatman filter paper no 1 (Whatman International Ltd., Maidstone, UK). Then, the extracted solvents were vaporized under lowered pressure and then air-dried. The extract was prepared at 1000 mg/mL in 100% dimethyl sulfoxide (DMSO) as a stock solution. Chlorhexidine (Sigma-Aldrich, Missouri, USA) was used as a positive control. The extracts and the antibiotics were dissolved in 100% DMSO and stored at 4°C until further use.

### 2.2. Parasite Culture

All experiments were conducted in compliance with the biosafety guidelines for scientific research at Walailak University, Nakhon Si Thammarat, Thailand (Ref. No. WU-IBC-66-020). *Acanthamoeba triangularis*-T4 WU19001 was cultured in a culture flask containing Peptone-Yeast Extract-Glucose (PYG) medium, as previously reported [7]. The medium contained 18 g glucose, 20 g proteose peptone, 2 g yeast extract, 0.98 g MgSO_4_ × 7H_2_O, 1 g sodium citrate dihydrate, 0.02 g Fe (NH_4_)_2_ (SO_4_)_2_ × 6H_2_O, 0.34 g KH_2_PO_4_, 0.355 g Na_2_HPO_4_ × 7H_2_O, and 1000 mL distilled water. The parasite samples were incubated at room temperature. Following incubation, trophozoites were harvested after 3 days, while cysts were collected after 14–21 days.

### 2.3. Preliminary Screening of Anti-*Acanthamoeba* Activity of the Extracts

Preliminary screening of the anti-*Acanthamoeba* activity of the plant extracts against the trophozoite and cyst of *A. triangularis* was carried out at a concentration of 1000 μg/mL. The trypan blue exclusion assay, as previously described [10], was employed for this purpose. Briefly, the trophozoites and cysts were rinsed twice with Page's saline solution (PAS) and centrifuged at 4000 rpm for 5 min. Then, an aliquot of 100 μL of the suspension of the trophozoites or cysts (2 × 10^5^ cells/mL) was added into 96-well plates, containing 100 μL of each extract at a concentration of 1000 μg/mL. One percent DMSO was used as a negative control because the final concentration of DMSO presented in the extract was 1%. Subsequently, the samples were incubated for 24 h at room temperature. The viability of *A. triangularis* was investigated by a trypan blue exclusion assay by observing the number of live (nonstained) and dead (stained) cells under an inverted microscope (Nikon, Tokyo, Japan). The relative percentage of growth inhibition was specified as

(1) $$\begin{array}{c} \text{Relative percentage of growth inhibition}=100-\frac{\text{mean of the treated parasite}}{\text{mean of the control}}*100. \end{array}$$

The plant extracts were selected when they showed ≥ 90% growth inhibition against the trophozoites and cysts, compared with the negative control.

### 2.4. Determination of Minimal Inhibitory Concentration (MIC)

The determination of the MIC values for the chosen plant extracts against *A. triangularis* was conducted using a broth microdilution assay, as described previously [7]. Briefly, 100 μL of a suspension containing trophozoites and cysts cultured in PYG medium (2 × 10^5^ cells/mL) was added into 96-well plates. Subsequently, 100 μL of plant extracts, successively diluted to concentrations ranging from 125 to 1,000 μg/mL, was added to the respective wells. As benchmarks, 1% DMSO and chlorhexidine were included as negative and positive controls, respectively. The samples were then incubated at room temperature for 24 h. The MIC value was defined as the lowest concentration of the plant extract that induced ≥ 90% growth inhibition (mean ± SD) in both trophozoites and cysts, as determined by trypan blue exclusion assay, relative to the control group. It was noticed that chloroform extract of *Knema retusa* wood showed the best anti-*Acanthamoeba* activity (Table 2). Therefore, the extract was chosen for further studies.

### 2.5. Time Kill Study of Kre Against Trophozoites and Cysts

Time-kill kinetics of Kre against *A. triangularis* trophozoites and cysts were investigated as described [11] with modifications. Briefly, the suspension of trophozoites and cysts cultured in PYG medium (2 × 10^5^ cells/mL) was treated with Kre at 1, 2, 4, and 8 times the MIC and incubated at room temperature. One percent DMSO was included as negative control. The samples were harvested at specific intervals: 0, 4, 8, 12, 16, 20, and 24 h. At each time point, *A. triangularis* viability was investigated using trypan blue exclusion assay. The experiment was carried out in triplicate, and the results were presented as mean log numbers of organisms ± standard deviation (SD).

### 2.6. Fluorescence Microscopy by Acridine Orange (AO) and Propidium Iodide (PI) Staining

The method by which *Acanthamoeba* cell death occurred following exposure to Kre was explored using an AO/PI double-staining assay, as described previously [12]. Briefly, *A. triangularis* trophozoites were treated with Kre and chlorhexidine in microcentrifuge tubes for 24 h. Then, samples were centrifuged at 4000 rpm for 5 min and washed twice with 0.01 M phosphate buffer solution (PBS). The cell pellets were suspended in a 100 μL AO/PI staining solution, which was prepared by adding 200 μL of AO (1 mg/mL) and 200 μL of PI (1 mg/mL) (Sigma Chemical Co., MO, USA) in 600 μL PBS solution. The samples were incubated for 10 min in the dark and placed on a slide, carefully covered with a coverslip. The trophozoites were visualized via a fluorescent microscope (Leica TCS SP5 laser scanning confocal microscope, Leica Microsystem Inc., Wetzlar, Germany). Live cells exhibited a green fluorescence (AO), while deceased cells displayed red fluorescence (PI). This AO/PI staining approach allowed for the differentiation between live and dead trophozoites based on their distinct fluorescence patterns. In addition, morphology of the treated *A. triangularis* trophozoites was observed under inverted microscope.

### 2.7. Scanning Electron Microscopy (SEM)

The morphology of the trophozoites and cysts after treatment with Kre was observed by SEM (Zeiss, Munich, Germany) as reported [7] with minor modifications. The trophozoites and cysts of *A. triangularis* were treated with the extracts at a concentration of 4 × MIC on a sterile glass cover slip in a 24-well plate and incubated at room temperature for 24 h. Then, specimens were washed twice with PBS and fixed with 2.5% glutaraldehyde in PBS at 4°C for 24 h. Subsequently, the samples were rinsed with PBS and dehydrated in a series of grades of ethanol (20%–100%). Then, the dehydration of the samples was further performed using a critical point dryer (CPD). After that, the specimens were put in a desiccator overnight. Then the samples were coated with gold particles. The morphological characteristics (size, shape, and structure) of *A. triangularis* post-treatment were observed under SEM.

### 2.8. Synergistic Effects of Kre and Chlorhexidine Against *A. triangularis*

The investigation into the synergistic effects of Kre in combination with chlorhexidine against *A. triangularis* trophozoites and cysts was carried out by a checkerboard assay, as described before [13] with minor modifications. Starting with the individual MICs of Kre and chlorhexidine, as determined in the prior stages, a checkerboard assay was conducted. This involved creating a grid of twofold dilutions for both Kre and chlorhexidine, intending to explore their combined effects. The growth inhibition of the combinations and the individual agents was determined by a trypan blue exclusion assay. The fractional inhibitory concentration index (FICI) was determined as follows:

(2) $$\begin{array}{c} \text{FICI}=\left(\frac{\text{MIC of Kre in Combination}}{\text{MIC of Kre alone}}\right)+\left(\frac{\text{MIC of Chlorhexadine Combination}}{\text{MIC of Chlorhexidine alone}}\right). \end{array}$$

The index was interpreted as follows.

FICI < 0.5 = synergism.

0.5 ≤ FIC < 1.0 = partial synergy.

FIC = 1.0 = additive.

FIC > 2.0 = antagonism.

### 2.9. Effects of Kre on Adhesion of *A*. *triangularis* to Polystyrene Plastic Surfaces and Contact Lenses

The activity of Kre on the adhesion of *A. triangularis* trophozoites was investigated on a 96-well polystyrene plate (0.33 cm^2^ of culture area, 0.075–0.2 mL of proposed working volume, VWR International, Missouri, USA) and contact lenses (Duna Plus, Singapore) as previously described [7]. Briefly, the trophozoites were cultured in PYG supplemented with Kre at sub-MICs. The positive and negative controls were chlorhexidine and 1% DMSO, respectively. The microtiter plates were incubated at 25°C for 24 h. Nonadhesive cells were eliminated by removing the old medium and washing twice with PAS. To detect the adhesion of the trophozoites on the 96-well plate, the samples were stained with 0.1% crystal violet for 30 min, washed twice with sterile distilled water, and air-dried overnight at room temperature. Then, 200 μL of DMSO was added to dissolve the stained cells. The plates were measured at an optical density of 570 nm. The activity of Kre on *A. triangularis* adhesion was calculated as follows:

(3) $$\begin{array}{c} \text{Relative percentage of adhesion}=\frac{\text{mean of the treated well}}{\text{mean of the control}}*100. \end{array}$$

To determine the adhesion of the trophozoites to contact lenses, the samples were dissolved in tubes holding 500 μL of PAS and mixed. The adhesive cells were then stained with trypan blue. The adhesive cells were detected under an inverted microscope (Nikon, Tokyo, Japan).

### 2.10. Removal of Adhesive *A. triangularis* Trophozoites on Polystyrene Surfaces and Contact Lenses by Kre

Removal of adhesive *A. triangularis* trophozoites on polystyrene surfaces and contact lenses by Kre was investigated as previously described [7] with minor modifications. Briefly, 100 μL of the trophozoites (2 × 10^5^ cells/mL) in PYG was inoculated in the 96-well plate and the contact lenses and incubated at room temperature for 48 h. Then, the old medium was removed, and the fresh medium containing Kre at 1/2–2 × MIC was added. The positive and negative controls were chlorhexidine and 1% DMSO, respectively. After that, the microtiter plates were incubated at 25°C for 24 h. To detect the removal of the trophozoites on the 96-well plate, the samples were measured by a crystal violet assay as described above. To investigate the removal of the trophozoites from the contact lenses, the samples were measured by a trypan blue exclusion assay as described above.

### 2.11. The Protein Three-Dimensional (3D) Structure Prediction

Computational modeling was used to investigate the influence of various substances on *Acanthamoeba* sp. essential proteins. The protein *A*. *triangularis* beta-tubulin (At-β-tubulin) is the subject of this study. The study examined the molecular docking relationship between *Acanthamoeba* tubulin and E2N, which is the primary constituent in Kre. The 3D structure of this protein was predicted using the SWISS-MODEL service since it lacks a crystal structure. Moreover, the initial input was established with the aid of At-β-tubulin FASTA sequence (GenBank: AFI57878.1), and the improvement of the quality of the projected 3D image was observed with ModRefiner [14]. Lastly, PROCHECK was utilized to evaluate the protein structures' stereochemical quality [15].

### 2.12. Ligand Binding Pocket Prediction

The ligand binding pockets were predicted using DeepSite, a protein binding pocket predictor based on deep neural networks [16]. The prepared protein structure was first input in Protein Data Bank (PDB) format. Next, the grid box center was established prior to the molecular docking procedure by taking into account the DeepSite prediction results.

### 2.13. Preparation of Protein and Ligand Structures for Molecular Docking

Prior to molecular docking, the protein structure was dehydrated in order to expose the amino acid residues. After that, nonpolar hydrogens were combined, polar hydrogens were added to amino acid residues, and the protein structure was given a Kollman charge. The data were saved in the PDBQT format (PDB, Partial Charge (Q), and Atom Type (T)) after stabilized protein structures were assigned partial charges and atom types. The ligand was prepared by merging nonpolar hydrogens and adding Gasteiger charges and polar hydrogens to the ligand structures. Finally, for stabilized ligand structures, the ligand structures were saved in the PDBQT format. After the receptor and ligand structures were built, AutoGrid4 software version 4.2 was used to build the grid maps that showed the system during the docking process. The grid box's center was ascertained using the DeepSite prediction results. The receptor assembly as a whole was included in the grid dimension, which measures 60 × 60 × 60 Å, with a 0.375 Å spacing. For every procedure, AutoDock Auxiliary Tool (ADT) Version 4.2 was utilized [17].

### 2.14. Molecular Docking of E2N Compounds to At-β-Tubulin

The molecular docking procedure was carried out using AutoDock4 Version 4.2 [17]. There were 50 GA runs in each docking stage, with 200-unit maximum population. As a result, a total energy evaluation of 2,500,000 units was needed for each dock. For every docking, the average elitism value was 1.00, the average cross-over rate was 0.80, and the average mutation rate was 0.02. The combination of local search (using Solis and Wets algorithm) and global search (using the Lamarckian Genetic Algorithm alone) was achieved. This parameter was used to perform 10,000 independent docking tests on each ligand. In order to ensure that the results were accurate, this procedure was carried out five times. Using the ADT Version 4.2, the inhibitor constant and the protein-ligand lowest binding energy (Δ*G* bind) were determined.

### 2.15. MDS and Trajectory Analysis

The binding stability, fluctuation, conformational changes, and kinetic behavior of specific drugs to the targeted protein were examined using a MDS [18, 19]. Before evaluating a drug molecule in an experimental lab, the dynamic simulation approach is frequently used to ascertain its properties. In order to examine the MDS of the target-ligand complex structure, we make use of Schrodinger's “Desmond v3.6 Program” (a commercial version that runs on Linux) [20]. The program solved the entire system using the TIP3P water model. For system preparation, the orthorhombic (box-shaped) form was taken into consideration, and a distance of 10 Å was kept from the box's wall. By adding appropriate ions, such as Na+ and Cl−, with a concentration of salt equal to 0.15 M, charges were electrically neutralized. After building the solvated system with the protein in complex with the ligand, the system was minimized and relaxed using the default protocol by using the OPLS-2005 force field settings within the Desmond module [21]. After 50 PS recording intervals with an energy of 1.2, NPT ensembles that used the Nose–Hoover temperature coupling and isotropic scaling approach were kept at 300 K and one atmospheric (1.01325 bar) pressure [22]. The quality assessment of the MDS and the simulation event was suggested by the simulation interaction diagram (SID) module in the Schrodinger program [23]. Furthermore, the stability and dynamic characteristics of these complexes were analyzed through the calculation of root mean square deviation (RMSD), root mean square fluctuation (RMSF), radius of gyration (Rg), solvent-accessible surface area (SASA), molecular surface area (MolSA), and polar surface area (PSA) values [24].

### 2.16. Statistical Analysis

All experiments were carried out three times. The statistical package Version 19 (SPSS Inc., Chicago, IL, USA) was used to analyze the data. In addition, the statistical analysis was examined using a two-tailed unpaired Student's *t*-test. The data were displayed as mean ± SD. According to the report, a difference that was deemed statistically significant was one where *p* < 0.05.

## 3. Results

### 3.1. Anti-*Acanthamoeba* Activity of the Extracts Against *A. triangularis*

As shown in Table 1, 44 plant extracts from 5 plant species demonstrated anti-*Acanthamoeba* activity against the trophozoites, and the cysts were determined at 1000 μg/mL. Consequently, the percent growth inhibition of the trophozoites treated with the extracts ranged from 59% to 100%, whereas against cysts, inhibition ranged from 47% to 100%. The extracts that showed ≥ 90% growth inhibition (mean ± SD) of trophozoites and cysts when compared with the control were selected for further study.

### 3.2. The MIC Values of the Selected Extracts Against *A. triangularis*

The extracts that showed ≥ 90% growth inhibition against the trophozoites and cysts were selected to determine the MIC values. As shown in Table 2, the selected plant extracts possessed strong anti-*Acanthamoeba* activity against both trophozoites and cysts. The MIC values of the extracts against trophozoites ranged from 31.25 to 1000 μg/mL, while, the MIC values of the extracts against cysts were 62.50–1000 μg/mL. A chloroform extract of Kre showed the strongest anti-*Acanthamoeba* activities against both trophozoites and cysts, with MIC values of 32.25 and 62.50 μg/mL, respectively. Hence, Kre was chosen for further studies. In addition, the MIC values of the positive control, chlorhexidine, against trophozoites and cysts were 15.62 and 62.50 μg/mL, respectively.

### 3.3. Killing Activity of Kre Against *A. triangularis*

**Figure 1:**
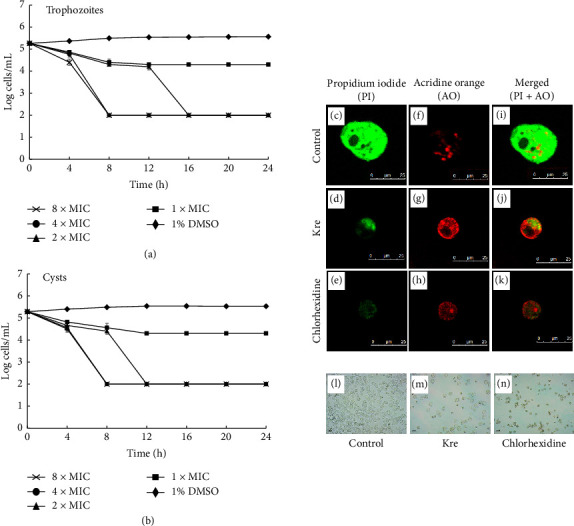
Killing activity of Kre against trophozoites and cysts as determined by time-kill study (a, b) and confocal microscope (c–k). One percent of DMSO and chlorhexidine were used as the negative and positive controls, respectively. The cells were then stained with propidium iodide (PI) and acridine orange (AO). Magnifications were revealed as 800x. Morphology of the trophozoites under inverted microscope (l–n). Magnifications were revealed as 200x.

The killing activity of Kre against *A. triangularis* was determined by a time kill kinetic study and confirmed by an AO/PI double-staining assay. Kre exhibited anti-*Acanthamoeba* activity that was concentration dependent, resulting in the reduction of cell viability of both the trophozoites and cysts (Figures 1(a) and 1(b)). Kre reduced the active cell concentrations by 3 logs within 8 h for both trophozoites and cysts at 4 × MIC and 8 × MIC. Furthermore, the viability of the parasite after treatment with Kre at 2 × MIC decreased by 3 logs in 12–16 h. However, Kre at 1 × MIC exhibited static effects against the parasite.

To confirm the killing activity of Kre, AO/PI fluorescence microscopy double staining was investigated to differentiate between viable and nonviable cells as well as the possible mode of cell death. The trophozoites exhibited green cytoplasm and bright green fluorescent of intact nucleus, indicating healthy and viable cells in the control groups (Figures 1(c), 1(f), and 1(i)). In contrast, the trophozoites treated with Kre (Figures 1(d), 1(g), and 1(j)) and chlorhexidine (Figures 1(e), 1(h), and 1(k)) showed orange to red granules in cells, indicating nonviable *Acanthamoeba* cells. It was noticed that the size of the trophozoites treated with both Kre and chlorhexidine was smaller than the control. Furthermore, the morphology of *A. triangularis* trophozoites treated with Kre (Figure 1(m)) and chlorhexidine (Figure 1(n)) as well as the negative control (Figure 1(l)) was demonstrated by inverted microscope.

### 3.4. Morphology of *A*. *triangularis* After Treatment With Kre

**Figure 2:**
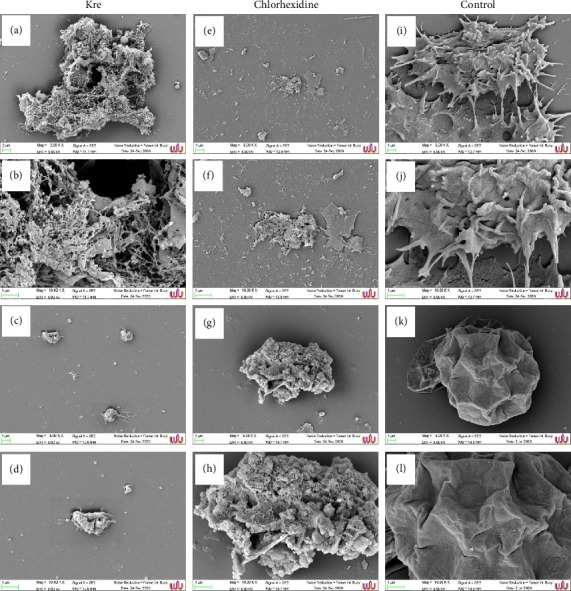
Morphology of *A. triangularis* trophozoites (a, b, e, f, i, j) and cysts (c, d, g, h, k, l) after treatment with Kre (a–d). The cells were treated with the extract at 4 × MIC (a–d). Chlorhexidine (e–h) and 1% DMSO (i–l) were used as positive and negative controls, respectively. The morphology of the parasite was observed by SEM. Magnifications were revealed as (a, c, e, g, i, k) = 5000x; (b, d, f, h, j, l) = 10,000x.

SEM images showed the morphology of trophozoites and cysts treated with Kre. In the control groups, amoeboid cells with many envelop spikes of the trophozoites were presented (Figures 2(i) and 2(j)). Moreover, the adhesion of the trophozoites to the surface by several long acanthopodia was observed. It was highlighted that disruption of the parasitic cells with abnormal shapes was observed when the trophozoites were treated with Kre (Figures 2(a) and 2(b)) and chlorhexidine (Figures 2(e) and 2(f)). Interestingly, the trophozoites had lost their mobility and began to shrink after treatment with both antimicrobial agents. Moreover, the treated trophozoites have lost robust acanthopodia.

The normal morphological characteristics of *A. triangularis* cysts with triangular shape and soft surface were presented in the control (Figures 2(k) and 2(l)). It has been highlighted that the cysts treated with Kre dramatically showed forms of retraction when compared to the control (Figures 2(c) and 2(d)). Disruption of the cyst wall was observed when the cysts were treated with Kre and chlorhexidine (Figures 2(g) and 2(h)).

### 3.5. Synergistic Effects of Kre and Chlorhexidine Against *A. triangularis*

**Figure 3:**
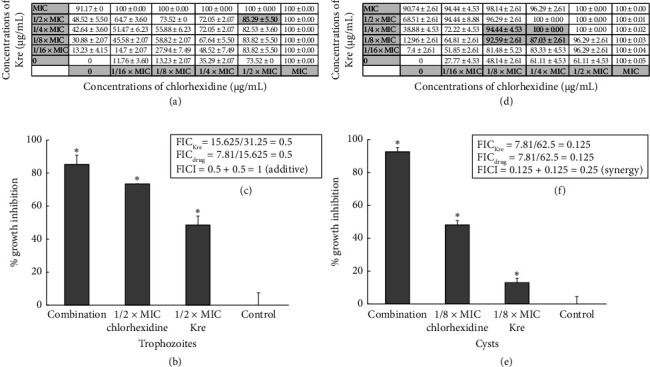
Synergistic effects of Kre in combination with chlorhexidine against *A. triangularis* trophozoites (a–c) and cysts (d–f). The parasites were treated with Kre or the drug alone or in combination (Kre + chlorhexidine) at different concentrations for 24 h. One percent of DMSO was used as a control. The data are presented mean ± SD (⁣^∗^significant difference; *p* < 0.05). Percent inhibition of the growth of the combination (a, d), percent growth inhibition of the representative combination (b, e), and FIC index (c, f) are presented.

According to the strong cyst walls that contained two layers, the synergistic effects of Kre and chlorhexidine against *A. triangularis* were determined. The combination of Kre at 1/8 × MIC and chlorhexidine at 1/8 × MIC showed synergistic effects against the cysts with a FICI of 0.25 (Figures 3(d), 3(e), and 3(f)). A significant inhibition of the trophozoites by the combination of Kre and chlorhexidine at 1/2 × MIC was observed. However, the combination against the trophozoites demonstrated additive activity with an FICI of 1.00 (Figures 3(a), 3(b), and 3(c)).

### 3.6. Kre Reduced the Adhesion of *A. triangularis* to Plastic Surfaces

**Figure 4:**
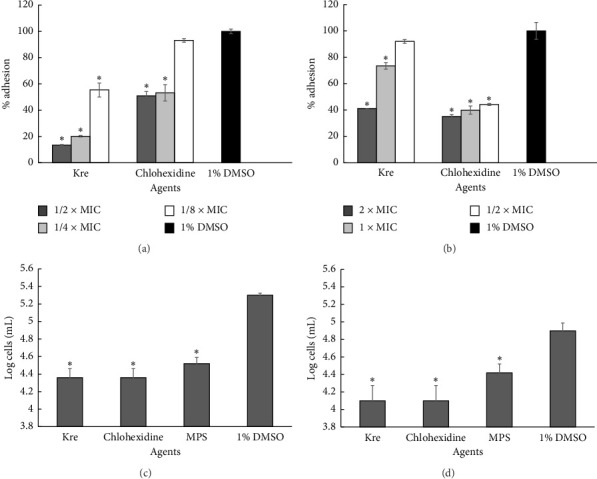
Effects of Kre on the adhesion (a, c) and removal (b, d) of *A. triangularis* trophozoites on the plastic surface (a, b) and contact lenses (c, d). Chlorhexidine and MPS were used as the positive controls, whereas 1% DMSO was used as the negative control (⁣^∗^significant difference; *p* < 0.05).

The reduction of the trophozoites on the surface was carried out by sub-MICs of Kre. The results revealed that Kre significantly inhibited the adhesion of *A. triangularis* trophozoites to the plastic surface, compared with the negative control (*p* < 0.05) (Figure 4(a)). Moreover, an 87% reduction in *Acanthamoeba* trophozoite adhesion to the surface was observed after treatment with 1/2 × MIC of Kre within 24 h. Chlorhexidine, the positive control, also inhibited the adhesion of the trophozoites and showed a percent inhibition of 49% compared with the control.

Since the trophozoites adhered to the plastic surfaces, we further determined the inhibitory activity of Kre to remove the trophozoites on the surface. The removal of a monolayer of trophozoites from the plastic surface was significantly detected by the treatment of both Kre and chlorhexidine (Figure 4(b)). It was found that 59% removal of the trophozoite adhesion to the surface was detected after treatment with Kre at 2 × MIC within 24 h. The percent elimination of chlorhexidine against the monolayer of trophozoites was 65%.

### 3.7. Kre Reduced the Adhesion of *A. triangularis* to the Contact Lens

According to the inhibitory activity of Kre against *A. triangularis* adhesion on the plastic surface, we further investigated the activity of Kre on the adhesion to contact lenses. The results revealed that the trophozoite adhesion was dramatically inhibited by Kre, chlorhexidine, and MPS at 1/2 × MIC (Figure 4(c)). Furthermore, 1 log cells/mL (90%) of trophozoites were reduced after treatment with Kre when compared with the control. Nearly 1 log cells/mL of the contact lens adhesive trophozoites were removed when the samples were treated with Kre (Figure 4(d)).

### 3.8. Protein 3D Structures and Ligand Binding Pocket Prediction

**Figure 5:**
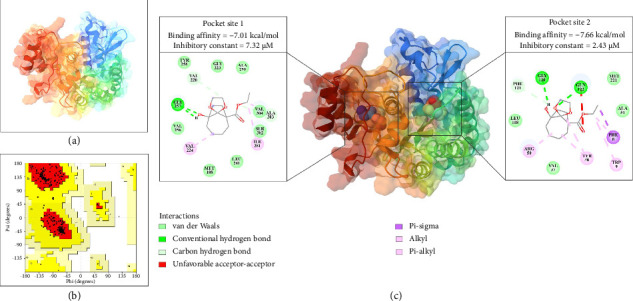
The predicted three-dimensional (3D) structures of the *A. triangularis* beta-tubulin containing 3D structure of the protein (a) and a Ramachandran plot of the protein (b). The interaction of the E2N compound with the *Acanthamoeba triangularis* beta-tubulin in two possible ligand-binding pockets (c).

The SWISS-MODEL service created a 3D structural model of At-β-tubulin using the tubulin beta-4B chain as a template.  Figure 5(a) illustrates the 3D structural model of At-β-tubulin. The sequence identity percentage with the template was 80.63%. The projected structure's QMEANDisCo Global was 0.75 ± 0.05. The Ramachandran plot of the At-β-tubulin model detected 94.2% of the residues in the most favored regions and 0.00% in the disallowed regions, showing that the generated protein structures had good stereochemical quality (Figure 5(b)).

The prediction of the ligand binding pockets was performed using DeepSite. The result showed two possible ligand-binding pockets throughout the protein. The first pocket was located at *x* center: 393.8, *y* center: 488.2, and *z* center: 447.8. The second pocket was located at *x* center: 415.8, *y* center: 482.2, and *z* center: 457.8 (Figure 5(c)).

### 3.9. Molecular Docking of E2N Compounds to At-β-Tubulin

AutoDock 4 was used for the molecular docking of E2N compounds toward At-β-tubulin proteins. With a binding energy (Δ*G* bind) of −7.01 kcal/mol and an inhibition constant (*K*_*i*_) of 7.32 μM, the compounds displayed good binding capability to the ligand binding pocket 1 of At-β-tubulin (Figure 5(c)). The compound forms a conventional hydrogen bond with the residue Gly257 (H-bond). Via a carbon H-bond, the compound interacts with the residues Val220 and Val304. Via van der Waals interactions, the compound interacts with the residues Met188, Gly223, Leu241, Val256, Tyr258, Ala259, and Ser302. Via alkyl, the compound interacts with the residues Val224, Ile281, and Ala303.

The compound also binds well to the ligand binding pocket 2 of At-β-tubulin, with a binding energy (Δ*G* bind) of −7.66 kcal/mol and an inhibitory constant (*K*_*i*_) of 2.43 μM. The compound forms a conventional hydrogen bond with the residues Gly120 and Gln122 (H-bond). Via a carbon H-bond, the compound interacts with the residue Phe121. Via van der Waals interactions, the compound interacts with the residues Val37, Ala51, Leu118, and Met221. The compound interacts with the Phe8 residue through pi-sigma. Via alkyl or pi-alkyl, the compound interacts with the residues Phe8, Trp9, Tyr38, and Arg50.

### 3.10. MDS

The stability and intermolecular interaction of the protein-ligand complex can be understood in reference time by using MDS in computer-aided drug discovery. In an artificial setting, it can also ascertain the complicated system's conformational change. In particular, RMSD, the radius of gyration (Rg), RMSF, SASA, and the total number of intra-molecular hydrogen bonds of the protein with the time-dependent function of MD were examined through the simulation trajectory in order to analyze the outcome of MDS. Using an all-atoms 100 ns MDS, the dynamic nature and interaction stability of the protein beta-tubulin (At-β-tubulin) bound with identified dietary compounds were investigated.

#### 3.10.1. RMSD Analysis

**Figure 6:**
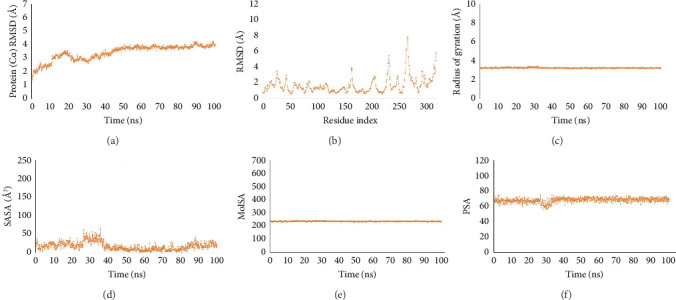
RMSD values (a), RMSF values (b), radius of gyration (Rg) values (c), SASA values (d), MolSA values (e), and PSA (f) values for the ligand molecule in complex with the protein beta-tubulin (At-β-tubulin) in 100 ns MDS assessments, where the selected ligand compound E2N (CID-85469100) bound.

When performing MDS, the root-mean-square error (RMSE), sometimes referred to as RMSD, is commonly used to calculate the mean value change caused by atom dislocation from a given frame to a reference frame. This information is useful in determining whether or not the simulation has equilibrated. Based on Cα atoms, the RMSD of the molecules when combined with beta-tubulin (At-β-tubulin) was calculated. When the average structural change from one-time frame to other stays within the range of 0.1–0.5 nm, the RMSD of complex structures is deemed acceptable. Therefore, a 100 ns MDS was run and the corresponding RMSD value was noted in order to assess the conformation change of the desired protein in the complex of the chosen drug candidate E2N (CID-85469100). For the compound E2N, the fluctuations increased at the start of the 0–35 ns MDS. After 35 ns of MDS time, the compound displayed an acceptable condition of equilibration (Figure 6(a)).

#### 3.10.2. RMSF Analysis

When describing a protein, the RMSF value is essential as it gives insight into the local alterations of the protein and the protein chain [25]. For the chosen natural compound E2N (CID-85469100), the RMSF was computed using the index of the residue Cα from the complex with beta-tubulin protein to track the modification in protein structural flexibility during the attachment of the chosen compound to a particular residual position, as illustrated in Figure 6.

Upon analyzing the RMSF graph, it was shown that the greatest variations for E2N occurred between residues 220 and 270, with a maximum fluctuation of 7.9 Å. It is noteworthy to point out that the free-floating of these areas led to a greater RMSF of terminal amino acids. Based on the above-average value and the discussion in conjunction with Figure 6(b), it is possible to conclude that all of the beta-tubulin's (At-β-tubulin) amino acids were stable and tightly bound to the molecules during the dynamic phases.

#### 3.10.3. Rg Analysis

To explain the rigidity of the complex in the MDS, any macromolecule attached to a small molecule must be stiff and compact. One statistical measure that may be used to show the rigidity and compactness of the molecules in the dynamic states is the Rg, which is derived from the trajectories of the MDS. As a result, Figure 6(c) displays an analysis of Rg over a 100 ns simulation time for medication candidate compound E2N, which has an Rg value of 3.2 in a complex with the desired protein beta-tubulin (At-β-tubulin). During the MDS, the complex appeared to be incredibly tight and compact. The low and steady deviation of the system was explained by the fact that not a single system was observed to have a bigger departure, which supported the folding of the protein in the dynamic phases.

#### 3.10.4. Analysis of SASA, MolSA, and PSA

The amount of SASA in biological macromolecules regulates their structure and functionality. Proteins' solvent-like characteristics (hydrophilic or hydrophobic) and protein-ligand interactions can be better understood by utilizing active sites and/or ligand-binding sites, which are frequently found on the surface of the protein. The SASA value of the compound (E2N) structure with the targeted protein beta-tubulin (At-β-tubulin) varied between 10 and 55 Å2, suggesting that high concentrations of the chosen ligand compound were present in the complex systems' amino acid residues (Figure 6(d)).

When the probe radius is adjusted to 1.4, the MolSA is equal to the van der Waals surface area.  Figure 6(e) illustrates that the therapeutic candidate chemical E2N, which targets the protein beta-tubulin (At-β-tubulin), has the typical van der Waals surface area in our *in silico* analysis. Furthermore, a structure's PSA is exclusively contributed to by the atoms of nitrogen and oxygen. In this case, the therapeutic candidate E2N that targets the beta-tubulin protein (At-β-tubulin) had a high PSA score (Figure 6(f)).

#### 3.10.5. Evaluation of Intramolecular Bonds

**Figure 7:**
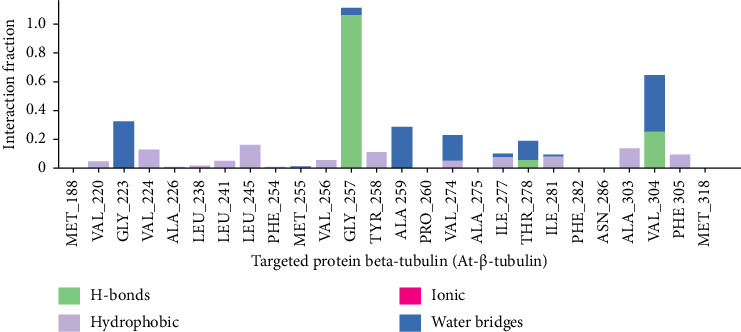
Interaction of the ligand E2N (CID-85469100) with the targeted protein beta-tubulin (At-β-tubulin). During the 100 ns simulation duration, the interactions with intramolecular bonds between protein and ligand were analyzed, as seen in the stacked bar charts.

The protein-ligand interaction's hydrogen bond, hydrophobic, ionic, and water bridge bond all have a significant impact on drug selectivity, metabolism, and adsorption. Holding the molecules inside the active site cavity is dependent on the hydrogen bonding between the ligand and the protein. The SID of the Schrodinger has been used to track protein interactions with the chosen chemical E2N. The stacked bar charts (Figure 7) display the hydrogen bond, hydrophobic, ionic, and water bridge interactions discovered during the interaction study. It was discovered that the molecule E2N (CID-85469100) forms several ionic, hydrogen, hydrophobic, and water bridge bonding interactions with beta-tubulin residues. The enhanced stability of ligands to the protein binding site is indicated by the study of the number of H-bonds created in the beta-tubulin-E2N complex.

## 4. Discussion

Severe vision loss and complete blindness in contact lens users are the main issues associated with the use of contact lenses contaminated with amoeba, including *A. triangularis*. It is challenging to eradicate *A. triangularis* using the host's immune system and medicines due to its robust pathogenesis in *Acanthamoeba* keratitis, including adhesion [26]. Therefore, preventing *A. triangularis* contamination of the contact lenses through the use of antimicrobial drugs may be an additional strategy to reduce the risk of infection. This investigation demonstrated Kre's amebicidal and antiadhesion properties against *A. triangularis* T4 on contact lenses and plastic surfaces.

As demonstrated by the MIC determination, time kill kinetic analysis, and fluorescence microscopy double staining assay, Kre exhibited potent anti-*Acanthamoeba* action against both trophozoites and cysts of *A. triangularis*. It is interesting to note that Kre showed strong anti-*Acanthamoeba* activity against both trophozoites and cysts, with MIC values that were very close to those of chlorhexidine, the recommended medication for treating *Acanthamoeba* infection. Our research team recently reported on the antibacterial, antiadhesion, and antibiofilm properties of Kre against isolates of *Staphylococcus aureus* and *Staphylococcus haemolyticus* from bovine mastitis [9]. Additionally, some plant species in the genus *Knema* showed antibacterial action against *Candida albicans* and *S. aureus*, including *Knema attenuata* seed and aril extracts [27]. As such, it is worthy of note that this is the first report on the anti-*Acanthamoeba* activity of Kre against *Acanthamoeba* spp.

We observed that the trophozoites treated with Kre and chlorhexidine had a much smaller size than the control group that was not treated. According to a prior study, chlorhexidine is positively charged and ionic with the amoeba's negatively charged plasma membrane; this results in cytoplasmic discontinuities, ionic leakage, structural and permeability alterations, and cellular destruction [28]. Membrane disruption–induced alterations in cell permeability and rupture can impact trophozoites and cysts' integrity, ultimately leading to the demise of parasite cells. In a similar vein, cationic carbosilane dendrimers demonstrated anti-*Acanthamoeba* efficacy against an *A. polyphaga* clinical isolate. As in this study, the chemical caused significant changes in *A. polyphaga* morphologies, such as rupture of the plasma membrane and smaller cells [29]. Furthermore, the cationic antiseptic polyhexamethylene biguanide interacts with the phospholipids in the membrane to alter its fluidity and structure, leading to ionic leakage and cell death [28]. *Acanthamoeba* cells that were not viable were also indicated by orange to red granules in the cells of the trophozoites treated with Kre and chlorhexidine, as seen by the staining of PI and AO. PI exhibits reddish-orange fluorescence in dead cells that have compromised membrane integrity [30]. Our results demonstrated that Kre showed synergistic effects in combination with chlorhexidine against the cysts. It is accepted that chlorhexidine acts as cationic molecule that binds to negatively charged microbial cell walls. However, the mechanisms of Kre in combination with chlorhexidine are recommended for further study.

Currently, the phytochemical constituents of Kre have been identified by our research group. In Kre, E2N was the most dominant compound with 64.83% peak area as detected by gas chromatography mass spectrometry [9]. This present study revealed that E2N can bind *in silico* to the tubulin molecule. Tubulins are monomers of microtubules that are widely recognized to be important in the maintenance of cell shape in various animals, such as *Acanthamoeba* species [5]. The results showed that E2N could bind to At-β-tubulin's ligand binding sites with good affinity, with an inhibition constant (Ki) of 2.43–7.32 μM and a binding energy (Δ*G* bind) of −7.1 to −7.66 kcal/mol. Based on this finding, the compound may potentially work against the parasite by targeting β-tubulin. Many herbicides, antimicrobials, and antineoplastics target tubulin, causing disruptions in the polymerization and depolymerization of tubulin [31]. Molecular modeling studies have demonstrated that the *Leishmania* β-tubulin gene mutation involves conformational changes, as evidenced by the elongation and twisting of the alpha helix structure and the displacement to the inside of one beta sheet's pocket [32]. The results of this investigation clearly show that Kre has antiadhesion activity against *A. triangularis* on contact lenses and plastic surfaces. These data are consistent with our earlier investigation [9] into the antiadhesion properties of Kre against *S. aureus* and *S. haemolyticus* isolated from bovine mastitis. Furthermore, antibiofilm actions as well as Kre's description of eliminating the staphylococci's developed biofilm have been reported [9]. Moreover, SarA, a key regulatory component associated with adhesion, is highly favored by E2N, the main component of Kre [9]. This study presents a contrast to the flat and adjacent trophozoites in the control, which were shown to adhere to the surface via many acanthopodia, and the treated trophozoites displayed shrunken cells without the acanthopodia. It has been documented that the trophozoites treated with curcumin [7], *Annona muricata*, and *Combretum trifoliatum* extracts [10] displayed a lump-like cystic form without the acanthopodia. However, Kre treated trophozoites did not show the lumpy shape of the cystic form. Acanthopodia are thought to be an organism's primary structure for adhering to surfaces such as contact lenses [2]. It is well known that the pathogenic *Acanthamoeba* with a high number of acanthopodia was found to be the virulence strain, whereas nonpathogenic parasites possessed fewer numbers of acanthopodia [33]. Moreover, a decrease in *Acanthamoeba* adhesion to the corneal epithelial cells occurred in the trophozoites without the acanthopodia [34]. Some medicinal plant extracts and plant-derived compounds, such as *Curcuma longa* extract, curcumin, *A*. *muricata* extract, and *C. trifoliatum* extract, reduced the adhesion of *A. triangularis* to the plastic surface and contact lens [7, 10]. More so, the treated trophozoites exhibited strong loss of acanthopodia and thorn-like projection pseudopodia that were similar to those in this present study. Therefore, loss of the acanthopodia by treatment with Kre could reduce adhesion to surfaces, including contact lenses. It is recommended that Kre be developed as a multiple solution for cleaning contact lenses. As shown in the results, the activity of Kre against trophozoite adhesion was better than that of commercial MPS.

According to observations, the primary component of Kre, E2N, exhibited high binding activity *in silico* against β-tubulin which is a monomer found in essential cytoskeleton components and also promoted organelle mobility in *Acanthamoeba* species. Since CADD can significantly lower the cost, time, and labor associated with the drug discovery process, it has emerged as one of the primary and essential tools for modern drug design. Understanding how a ligand behaves and binds to a particular target molecule has become simpler owing to the CADD method. An essential technique for CADD is MDS, which analyzes the biomolecular structures of proteins and ligands and gives a sense of how stable the protein-ligand interaction is [25]. Based on factors such as protein-ligand interaction, Rg, RMSD, and RMSF value, the trajectory files from the simulation were examined and demonstrated the stability of our lead chemical [35]. The compounds with the highest stability are indicated by the complex systems' RMSD values, while the protein-ligand complex's compactness is determined by the mean fluctuation measured by the RMSF values [36, 37].

The best stability of the compounds is shown by lower RMSD values, and the compactness of the protein-ligand complex is determined by the mean palpitation measured by RMSF values [38]. To understand more about the selected compound, the Rg, SASA, MolSA, PSA, and protein-ligand interaction were assessed. Consequently, the protein beta-tubulin (At-β-tubulin) has the lowest RMSD and RMSF values when compared to the compound that was chosen, according to MDS [18, 19]. The displacement of the center of mass from the two protein terminals is computed using the Rg [39]. Consequently, this measure provides a more comprehensive understanding of the folding features of proteins and checks the stability of the protein structure. A smaller Rg value indicates that the chemicals were disassociated from the protein, whereas a bigger value and high compactness were indicated. For a better understanding of the interaction between the protein-ligand complex, the SASA value is studied [40]. Total energy by region of the ligand and protein is used to measure the interaction of the entire protein surface with its water molecules. A more stable structure is indicated by a bigger SASA value, while a more densely connected combination of water molecules and amino acid residues is indicated by a lower value [41]. Using the protein beta-tubulin, the study discovered lower Rg and SASA values of the chosen chemical E2N. The drug's good potential is further supported by the MolSA and PSA graphs. Further evaluation of the compound with predicted values revealed a positive and stable binding result based on protein-ligand contact and hydrogen bond interaction [42]. Our research indicates that the highest energetically chosen chemical is coupled with a catalytic residue, which is necessary to inhibit beta-tubulin (At-β-tubulin).

Limitations of this study include the isolation of pure compounds (e.g., E2N) from Kre. Subsequently, the anti-*Acanthamoeba* mechanisms of action of Kre and its pure compounds against *A. triangularis* should be determined. Further study such as ex vivo analysis or nanoformulation to provide insight on the mechanism of Kre and the pure compounds against the trophozoites and cysts is therefore recommended. Importantly, the cytotoxicity of Kre on corneal epithelial cells should be explored.

## 5. Conclusion

In summary, Kre exhibited amebicidal activity against the *A. triangularis* T4 genotype with MIC values of 32.25–62.50 μg/mL. In addition, synergistic effects of Kre in combination with chlorhexidine against the cysts were also detected. A reduction in the 3-log viability of the parasite was observed after the treatment at 4 × MIC within 8 h. Interestingly, the trophozoites had lost their robust acanthopodia and began to shrink, while cyst wall disruption and dramatic forms of retraction were observed after treatment with the extract. Kre significantly inhibited and removed the adhesion of *A. triangularis* trophozoites to the plastic surface and contact lens. Impressively, 1 log cells/mL (90%) of the contact lens adhesive trophozoites were reduced and removed after treatment with Kre. E2N, the main compound presented in Kre, could bind well to the ligand binding sites of At- β-tubulin, with a binding energy (ΔG bind) of −7.-1 to −7.66 kcal/mol and an inhibitory constant (Ki) of 2.43–7.32 μM. It can be seen that the compound generated multiple connections via hydrogen, hydrophobic, ionic, and water bridge bonding and maintained these connections until the simulation was completed, facilitating the creation of a stable binding with the targeted protein. This information implies that Kre may have amebicidal and antiadhesion activities that can be used in the management or prevention of *A. triangularis* adhesion to contact lenses.

## Figures and Tables

**Table 1:** Preliminary screening of the plant extracts against *Acanthamoeba triangularis* T4.

Plant species	Part used	Solvents	% growth inhibition (mean ± SD)	
			Trophozoites	Cysts
*Burkillanthus malaccensis*	Bark	Chloroform	86.11 ± 1.96	67.16 ± 7.61
	Bark	Hexane	96.73 ± 0.00	89.55 ± 5.58
	Flesh	Chloroform	91.30 ± 1.53	89.55 ± 2.11
	Flesh	Methanol	59.78 ± 4.06	92.53 ± 2.11
	Leaves	Chloroform	85.86 ± 3.07	68.65 ± 3.65
	Leaves	Hexane	93.47 ± 2.66	91.04 ± 3.65
	Leaves	Methanol	95.65 ± 1.53	52.23 ± 5.58
	Peel	Chloroform	95.65 ± 1.53	67.16 ± 7.61
	Peel	Hexane	100.00 ± 0.00	100.00 ± 0.00
	Seed	Hexane	81.94 ± 5.19	83.58 ± 23.21
	Seed	Methanol	70.83 ± 3.40	95.52 ± 3.65
	Seed	Chloroform	100.00 ± 0.00	95.52 ± 3.65
	Wood	Chloroform	88.04 ± 4.06	61.19 ± 2.11
	Wood	Hexane	78.80 ± 6.91	74.07 ± 6.92
	Wood	Methanol	80.43 ± 2.66	91.04 ± 3.65

*Cleistanthus bracteosus*	Bark	Chloroform	78.26 ± 1.53	74.07 ± 4.53
	Bark	Hexane	80.43 ± 2.66	71.64 ± 5.58
	Bark	Methanol	89.13 ± 3.07	50.74 ± 3.65
	Leaves	Chloroform	81.52 ± 1.53	80.59 ± 5.58
	Leaves	Hexane	71.73 ± 3.07	82.08 ± 6.33
	Leaves	Methanol	67.39 ± 5.32	77.77 ± 4.53

*Diospyros hasselti*	Flesh	Chloroform	89.13 ± 3.07	70.14 ± 4.22
	Flesh	Hexane	91.30 ± 4.06	74.62 ± 2.11
	Flesh	Methanol	95.65 ± 1.53	59.70 ± 3.65
	Leaves	Chloroform	84.78 ± 1.53	65.67 ± 5.58
	Leaves	Hexane	79.34 ± 6.70	70.14 ± 2.11
	Leaves	Methanol	75.00 ± 1.53	85.07 ± 2.11

*Knema retusa*	Bark	Chloroform	89.13 ± 1.53	88.05 ± 2.11
	Bark	Hexane	80.43 ± 4.61	67.16 ± 5.58
	Bark	Methanol	93.47 ± 2.66	74.62 ± 5.58
	Leaves	Chloroform	91.30 ± 3.07	80.59 ± 4.22
	Leaves	Hexane	92.39 ± 4.06	97.01 ± 2.11
	Wood	Chloroform	100.00 ± 0.00	100.00 ± 0.00
	Wood	Hexane	96.73 ± 0.00	70.14 ± 7.61
	Wood	Methanol	89.13 ± 1.53	88.05 ± 4.22

*Litsea spathacea*	Bark	Chloroform	89.13 ± 1.53	64.17 ± 3.65
	Bark	Hexane	96.73 ± 0.00	47.76 ± 5.58
	Bark	Methanol	82.60 ± 1.53	73.13 ± 7.31
	Leaves	Chloroform	69.56 ± 3.07	64.17 ± 3.65
	Leaves	Hexane	90.21 ± 0.00	89.55 ± 2.11
	Leaves	Methanol	84.78 ± 7.68	65.67 ± 5.58
	Wood	Chloroform	89.13 ± 1.53	58.20 ± 4.22
	Wood	Hexane	90.21 ± 4.61	59.70 ± 3.65
	Wood	Methanol	90.21 ± 2.66	70.14 ± 4.22

**Table 2:** Minimal inhibitory concentration of the plant extracts against *Acanthamoeba triangularis*.

Plant species	Part used	Solvents	MIC (μg/mL)	
			Trophozoites	Cysts
*Burkillanthus malaccensis*	Bark	Hexane	1000	1000
	Flesh	Chloroform	1000	1000
	Flesh	Methanol	ND	1000
	Leaves	Hexane	1000	ND
	Leaves	Methanol	1000	ND
	Peel	Chloroform	500	ND
	Peel	Hexane	500	500
	Seed	Methanol	ND	1000
	Seed	Chloroform	250	500
	Wood	Chloroform	1000	ND
	Wood	Methanol	ND	1000

*Cleistanthus bracteosus*	Bark	Methanol	1000	ND

*Diospyros hasselti*	Flesh	Chloroform	1000	ND
	Flesh	Hexane	1000	ND
	Flesh	Methanol	500	ND

*Knema retusa*	Bark	Chloroform	1000	1000
	Bark	Methanol	1000	ND
	Leaves	Chloroform	1000	ND
	Leaves	Hexane	1000	1000
	Wood	Chloroform	31.25	62.50
	Wood	Hexane	500	ND
	Wood	Methanol	1000	1000

*Litsea spathacea*	Bark	Chloroform	500	ND
	Bark	Hexane	1000	ND
	Leaves	Hexane	1000	1000
	Leaves	Methanol	500	ND
	Wood	Chloroform	500	ND
	Wood	Hexane	1000	ND
	Wood	Methanol	1000	ND

Chlorhexidine	—	—	15.62	62.5

## Data Availability

The data will be made available upon reasonable request through the corresponding author.
