# Correction to “Amebicidal and Antiadhesion Activities of *Knema retusa* Extract Against *Acanthamoeba triangularis* T4 Genotype on Contact Lenses and Modeling Simulation of Its Main Compound, E2N, Against *Acanthamoeba* Beta-Tubulin”

**DOI:** 10.1155/sci5/9847010

**Published:** 2025-11-14

W. Mitsuwan, I. Sama-ae, S. Sangkanu, et al., “Amebicidal and Antiadhesion Activities of *Knema retusa* Extract Against *Acanthamoeba triangularis* T4 Genotype on Contact Lenses and Modeling Simulation of Its Main Compound, E2N, Against *Acanthamoeba* Beta-Tubulin,” *Scientifica* 2025 (2025): 4311313, https://doi.org/10.1155/sci5/4311313.

In the article, the following affiliation listed for Sonia M. Rodrigues Oliveira and Maria de Lourdes Pereira was incorrect:

• “Department of Medical Sciences, CICECO-Aveiro Institute of Materials, University of Aveiro, Aveiro, Portugal”

The correct affiliations for Maria de Lourde's Pereira are

• CICECO-Aveiro Institute of Materials, University of Aveiro, 3810-193 Aveiro, Portugal
• Department of Medical Sciences, University of Aveiro, 3810-193 Aveiro, Portugal

The correct affiliations for Sonia M. Rodrigues Oliveira are

• CICECO-Aveiro Institute of Materials, University of Aveiro, 3810-193 Aveiro, Portugal
• Universidade Católica Portuguesa, Faculty of Dental Medicine, Center for Interdisciplinary Research in Health (CIIS), 3504-505 Viseu, Portugal

The author list and affiliations have been corrected in the article accordingly.

We apologize for these errors.

